# Neurodegeneration with Brain Iron Accumulation in an Eleven-Year-Old Jamaican Male

**DOI:** 10.1155/2014/858056

**Published:** 2014-01-28

**Authors:** Peter Johnson, Roxanne Melbourne-Chambers, Nilesh Desai, Emma Greenaway

**Affiliations:** ^1^Department of Surgery, Radiology, Anaesthetics and Intensive Care, Faculty of Medical Sciences, University of the West Indies (Mona Campus), Kingston, Jamaica; ^2^Department of Child and Adolescent Health, Faculty of Medical Sciences, University of the West Indies (Mona Campus), Kingston, Jamaica; ^3^Department of Radiology and Imaging Sciences, Emory University School of Medicine, Emory University Hospital, BG21, 1364 Clifton Road, Atlanta, GA 30322, USA

## Abstract

We present a case of an eleven-year-old boy presenting with progressive extrapyramidal signs and dementia. His imaging findings demonstrated the classic eye-of-the-tiger sign on T2W magnetic resonance imaging. He was diagnosed with pantothenate kinase-associated neurodegeneration (PKAN). This is a rare autosomal recessive inborn error of coenzyme A metabolism, caused by mutations in *PANK2*. This is the first reported case of PKAN from the Caribbean.

## 1. Introduction

Pantothenate kinase-associated neurodegeneration (PKAN) is a rare autosomal recessive inborn error of coenzyme A metabolism, caused by mutations in *PANK2*. Clinical features include progressive extrapyramidal signs, pigmentary retinopathy, or optic atrophy and acanthocytosis [1]. Classic imaging findings which help establish the diagnosis include T2 weighted MRI brain features of bilateral anteromedial hyperintensity surrounded by a region of hypointensity in the medial globus pallidus (eye-of-the-tiger sign).

## 2. Case Report

An eleven-year-old boy presented to the University Hospital of the West Indies, Jamaica, with a history of neurodevelopmental regression with onset at the age of seven when he ceased speaking. By the age of 10 he was unable to sit unsupported or to self-care. He displayed aggressive behavior and dystonic posturing of the limbs, trunk, and the oromandibular region which subsided with sleep. He later developed swallowing difficulty. There was no family history of consanguinity or neurological disease and the perinatal period was normal.

Examination revealed an agitated boy who communicated using gestures and obeyed simple commands. His weight and height were <5th centile; the head circumference was normal. There were minor dysmorphic features, generalized muscle wasting, and limb contractures. Fundoscopy revealed bilateral disc pallor and mid-peripheral hyperpigmentation with bony spicules. The gag reflex was weak. Tongue thrusting, forceful jaw opening, and axial and appendicular dystonia were noted. He was noted to use the hand to forcibly close his jaw. There was weakness at the wrists and ankles. Deep tendon reflexes were normal.

**Figure 1:**
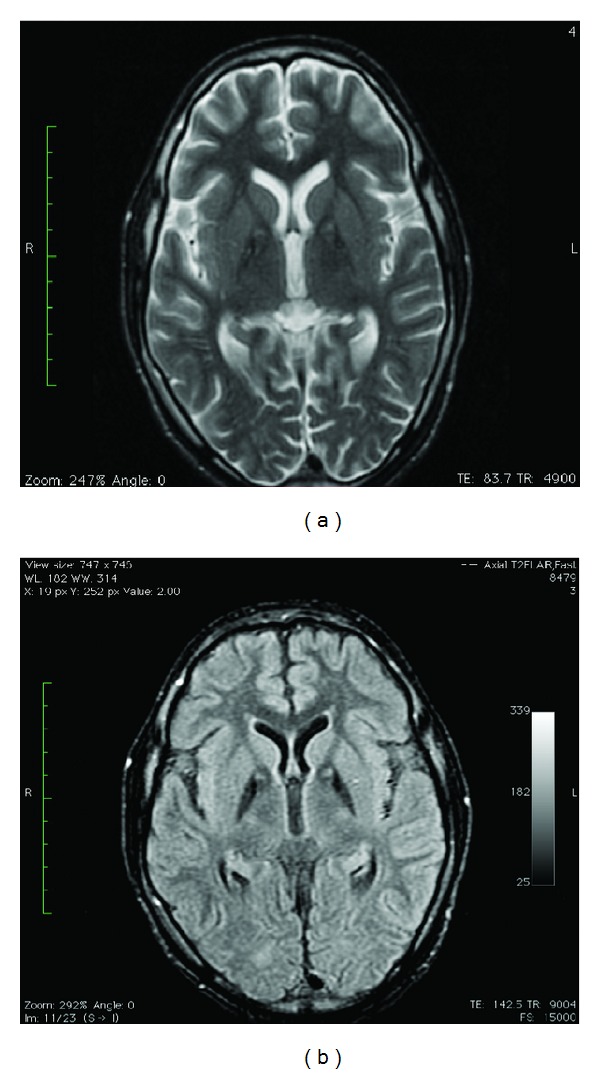
(a) Axial T2W fse. (b) Axial T2 FLAIR. Both demonstrate T2 shortening (hypointensity) with foci of T2 prolongation (hyperintensity) at the anteromedial aspect in the bilateral globus pallidus (eye-of-the-tiger sign).

On laboratory examination, there was mild elevation of hepatic transaminases. Ceruloplasmin, serum, and urinary copper levels were normal. Magnetic resonance imaging (MRI) of the brain demonstrated symmetric changes of homogenous T2 hypointensity in both globus pallidi. In addition, there were discrete foci of T2 hyperintensity at the anteromedial aspect in the bilateral globus pallidi. This appearance is commonly referred to as the “eye of the tiger” sign. The brain parenchyma was otherwise normal (Figure 1).

He was diagnosed with neurodegeneration associated with brain iron accumulation (NABI), also referred to as pantothenate kinase-associated neurodegeneration (PKAN). Molecular confirmation was not possible. He was treated with L-dopa/carbidopa and experienced marked reduction of the dystonia, improved hand eye coordination, and reduced dystonia. A gastrostomy was performed for enteral feeding.

## 3. Discussion

This is the first reported case of NABI from the Caribbean region. PKAN, a rare autosomal recessive inborn error of coenzyme A metabolism, caused by mutations in *PANK2 *was the most likely diagnosis. PKAN was first described in a sibship of five persons with dystonia, choreoathetosis, rigidity, and progressive dementia in 1922 [2]. Initially it was termed as Hallervorden-Spatz syndrome after the investigators originally described it [2]. This name has, however, since been discouraged due to the admissions of both investigators in using brains of executed prisoners in Nazi war camps [3]. Diagnostic criteria including onset in the first three decades, progression of symptoms and signs, extra-pyramidal dysfunction, and T2 weighted MRI brain features of bilateral anteromedial hyperintensity surrounded by a region of hypointensity in the medial globus pallidus (eye-of-the-tiger sign) have been proposed [1]. Corroborative features include corticospinal tract involvement, pigmentary retinopathy or optic atrophy, family history suggestive of autosomal recessive inheritance, and acanthocytosis, most of which were present in our patient. The clue to the diagnosis was the striking oromandibular dystonia, which is characteristic of the disease [4]. INAD presents in the first two years of life with psychomotor regression, gait instability, and optic atrophy by early childhood [5]. Atypical NAD presents at an average age of 4 years with gait instability, ataxia, diminished social interaction, and speech delay [5]. The eye-of-the-tiger sign, a central region of hyperintensity in the globus pallidus with surrounding hypointensity on T2 weighted imaging, is pathognomic of PKAN and is not present in INAD and atypical NAD, although the latter may have T2 hypointensity in the globus pallidus [6].

In summary, NBIA should be considered in the differential diagnosis of neurodegenerative disease associated with dystonia. Oromandibular dystonia is highly suggestive of this disorder. Although molecular confirmation was not possible for this case, neuroimaging along with clinical features provided an adequate diagnosis.
